# Diagnosis of α-thalassaemia by colorimetric gap loop mediated isothermal amplification

**DOI:** 10.1038/s41598-023-36676-2

**Published:** 2023-06-13

**Authors:** Worakawee Chumworathayee, Thongperm Munkongdee, Nattrika Buasuwan, Pornthip Chaichompoo, Saovaros Svasti

**Affiliations:** 1 Graduate Program in Molecular Medicine, Faculty of Science, Mahidol University, Bangkok, Thailand; 2 Thalassemia Research Center, Institute of Molecular Biosciences, Mahidol University, Salaya, Nakhon Pathom Thailand; 3 Division of Biology, Faculty of Science and Technology, Rajamangala University of Technology, Thanyaburi, Pathumthani Thailand; 4 Department of Pathobiology, Faculty of Science, Mahidol University, Bangkok, Thailand; 5 Department of Biochemistry, Faculty of Science, Mahidol University, Bangkok, Thailand

**Keywords:** Diagnostic markers, Genetics research, Biochemistry, Anaemia, PCR-based techniques

## Abstract

α-Thalassaemia is an inherited haemoglobin disorder that results from the defective synthesis of α-globin protein. Couples whom both carry the α-thalassaemia 1 gene are at risk of having a foetus with the most severe thalassaemia, Hb Bart’s hydrops fetalis, with a risk of maternal mortality. However, haematological parameters alone cannot distinguish between a α-thalassaemia 1 carrier and a homozygous α-thalassaemia 2, in which one α-globin gene has been deleted on each chromosome. A rapid and accurate molecular detection assay is essential for prevention of the disease in populations where α-thalassaemia 1 is common. Multiplex Gap-PCR analysis is widely used for diagnosis of α-thalassaemia. However, the technique requires a thermocycler and post-amplification processing, which limits its application in primary care or in rural areas in developing countries. Loop mediated isothermal amplification (LAMP) amplifies target DNA at a constant temperature and does not require a thermocycler. This study developed a colorimetric Gap-LAMP using malachite green to allow naked eye visualization of two deletional α-thalassaemia 1 commonly found in Asian populations, the Southeast Asian type (--^SEA^) and the Thai type (--^THAI^) deletions. The Gap-LAMP was performed on DNA samples from 410 individuals carrying various α-thalassaemia gene defects with 100% concordance with conventional Gap-PCR analysis. This method eliminates post-amplification processing or the use of expensive sophisticated equipment and allows screening large populations for the prevention and control of α-thalassaemia.

## Introduction

Hb Bart’s hydrops fetalis, homozygous α-thalassaemia 1, is the most severe thalassaemia, and all of these foetuses die either *in utero* or soon after birth with severe anaemia and tissue hypoxia. Importantly, the mother of the affected foetus has an increased risk of obstetric complications, including severe preeclampsia, dystocia and postpartum haemorrhage due to placentomegaly. Maternal mortality is estimated at nearly 50% if there is no medical treatment^1,2^. Affected pregnancies are usually terminated because of the associated maternal and perinatal morbidities. Identification of couples at risk, genetic counseling and early prenatal diagnosis is essential for an early decision to avoid the serious complications in late gestation. Hence, accurate diagnosis in population screening is important for preventing and controlling the disease. α-Thalassaemia is most often caused by deletion of α-globin genes and consequently reduced or absent α-globin chain synthesis. Two copies of the α-globin gene located on each homologous chromosome 16 contribute to the foetal (α_2_γ_2_) and adult (α_2_β_2_) haemoglobin. Thus, a variable number of α-globin gene defects lead to varying clinical manifestations in foetuses and adults. The most severe form of α-globin gene abnormality is α-thalassaemia 1, in which two copies of α-globin genes are *cis*-deleted from the same chromosome resulting in the absence of α-globin chain production from that chromosome. The α-thalassaemia 1 genotype is prevalent in Southern China and Southeast Asia. In China, the highest distribution of α-thalassaemia with prevalence in the range 4.64 to 7.91% was found in the southern provinces^3–5^. In Southeast Asia, the highest α-thalassaemia 1 prevalence was observed in Thailand (14.40%), followed by Laos (13.87%), Vietnam (4.61%), Malaysia (3.85%), Cambodia (1.96%) and Myanmar (1.86%)^6–11^. The most common type of α-thalassaemia 1 in the Asian population is the Southeast Asian type deletion (--^SEA^, SEA). In addition, another type of α-thalassaemia 1, the Thai type deletion (--^THAI^, THAI), has also been reported in Southern China and Thailand^3,5,8^.

Conventional screening for α-thalassaemia trait is based on an assessment of complete blood count (CBC), red cell morphology, measurement of haemoglobin levels, and haemoglobin type classification. While Hb typing can be used for α-thalassaemia disease diagnosis, it cannot diagnose α-thalassaemia trait as the result is the same as those of normal individuals. The α-thalassaemia 1 trait shows circulating red blood cells as hypochromic microcytes, with haematological parameters similar to homozygous α-thalassaemia 2, in which only one α-globin gene deleted on each chromosome. Therefore, only DNA-based methods can be used to solve this diagnosis limitation. A widely used technique for deletional α-thalassaemia diagnosis is multiplex Gap-PCR analysis which is based on primers designed to amplify DNA across the breakpoint region of the deleted gene^12^. Although the method gives cost effective and unequivocal results, this technique requires a thermocycler and a post-amplification process. The use of expensive and specialized equipment limits its application in primary care or in rural areas in developing countries. Loop mediated isothermal amplification (LAMP) amplifies target DNA by employing a strand displacement *Bst* DNA polymerase and four primers that recognize six distinct sequences of the target DNA for increased specificity^13^. The amplification reaction occurs at 60-65°C, allowing the use of a simple water bath or heat block. The LAMP technique has been widely used to diagnose a wide range of infectious diseases^14,15^, pharmacogenetic and disease risk alleles^16,17^ and genetic diseases^18–20^. In addition, the amplification products can be visualization using pH-sensitive dyes^21^, a fluorescent metal indicator^22^, fluorescent and non-fluorescent DNA intercalating dye^23,24^.

In order to cover the large populations of Southern China and Southeast Asia for screening couples at risk of having Hb Bart’s hydrops fetalis, accurate screening in primary care in rural areas without sophisticated equipment is needed. Herein, we combine the features of Gap-PCR and LAMP for a rapid and reliable diagnosis of α-thalassaemia 1 based on a colorimetric Gap-LAMP technique using the DNA intercalating dye malachite green, which allows for naked eye visualization. This technique has high sensitivity, specificity, accuracy, a rapid turnaround time and does not require specialized equipment. This newly developed method could be helpful for high-throughput screening in primary care facilities and aid in the prevention and control of the disease.

## Results

### Detection of deletional α-thalassaemia 1 gene by Gap-LAMP

**Figure 1:**
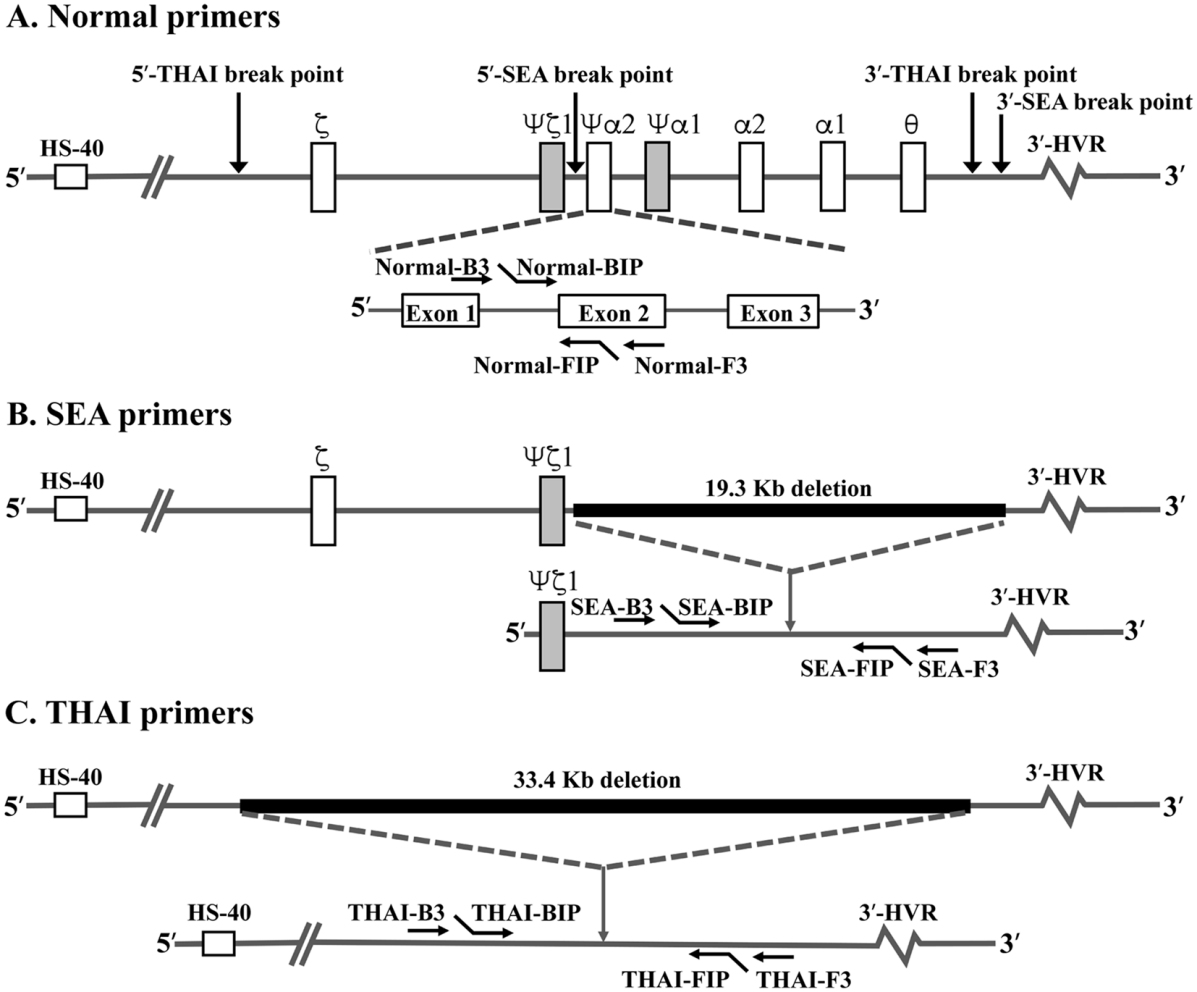
Positions of the primers used in Gap-LAMP. Schematic representation of the α-globin gene cluster and relative positions of the primers used in Gap-LAMP, (**A**) Normal primers, (**B**) SEA primers and (**C**) THAI primers. The black boxes indicate the extent of the SEA and THAI deletions.

**Figure 2:**
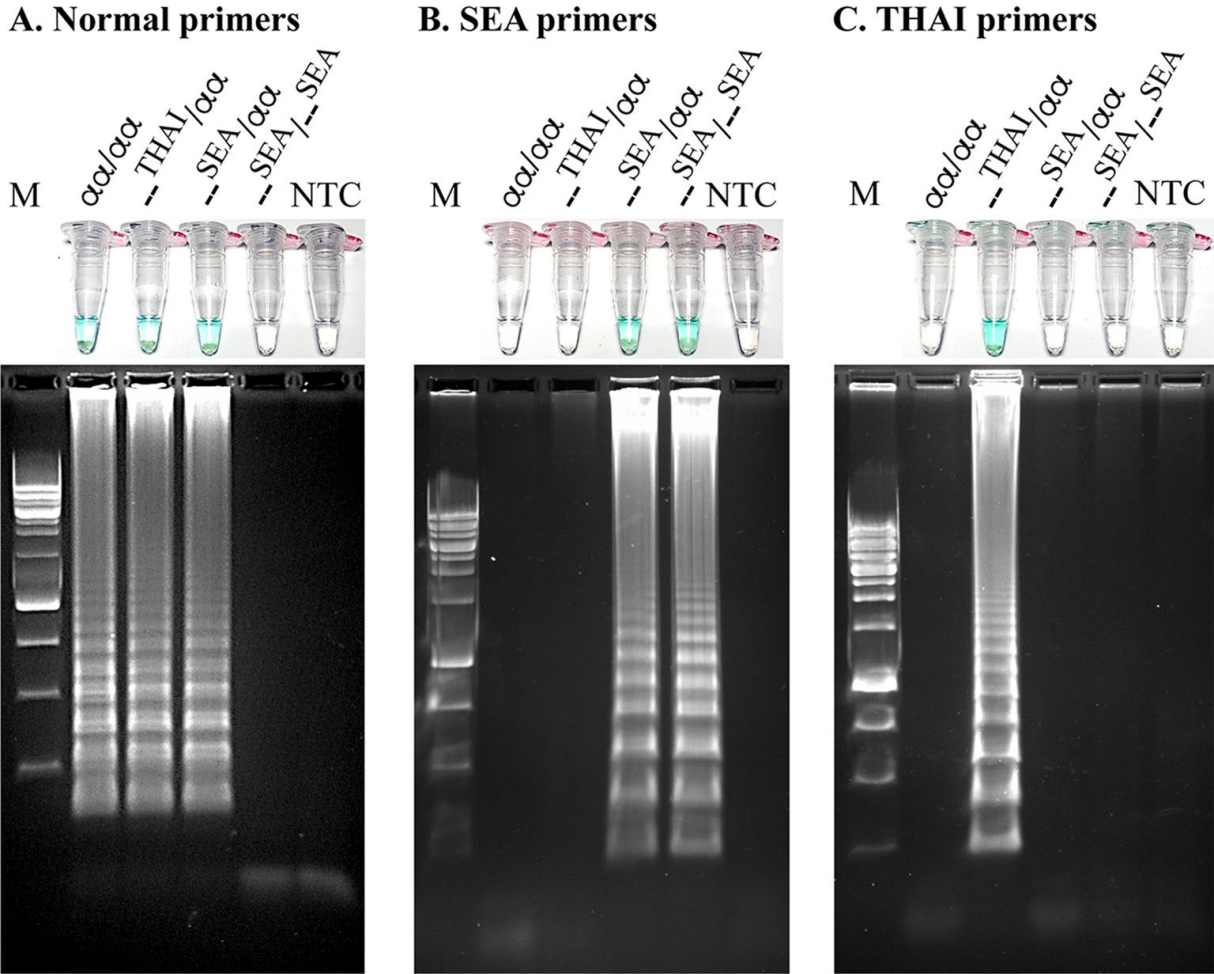
Specific amplification of deletional α-thalassaemia 1 gene by Gap-LAMP. Specific amplification of each Gap-LAMP primer sets, (**A**) normal primers, (**B**) SEA primers and (**C**) THAI primers, was demonstrated by amplification of genomic DNA from normal subject (αα/αα), α-thalassaemia 1 traits including (--^THAI^/αα) and (--^SEA^/αα), and HbBart’s hydrops fetalis (--^SEA^/--^SEA^). Visual appearance of colorimetric malachite green based Gap-LAMP reaction products (upper) and agarose gel electrophoresis (lower) are consistant and show specific amplification of each primer set. M, 1 kb DNA ladder; NTC, non-template control.

The LAMP reactions were first optimized and assessed on genomic DNA from individuals carrying known α-thalassaemia 1 genes. Three specific primer sets were used for amplification of α-thalassaemia 1 (SEA and THAI) and the normal gene (ψα2-globin gene) (Fig. 1 and Table 1). The Gap-LAMP primers had high specificity to distinguish between normal and the two deletional α-thalassaemia 1 (Fig. 2). With the normal primer set, a blue color solution indicating a positive reaction was observed after amplification of genomic DNA from a normal subject (αα/αα), SEA trait (--^SEA^/αα) and THAI trait (--^THAI^/αα), while the solution turned colorless (no amplification) when Hb Bart’s hydrops fetalis DNA (--^SEA^ /--^SEA^) was used as the genomic material (Fig. 2A). Importantly, the SEA primer set could amplify only SEA trait and Hb Bart’s hydrops fetalis as shown in Fig. 2B. In addition, the THAI primer set could amplify only THAI trait while negative reaction in normal subject, SEA trait and Hb Bart’s hydrops fetalis (--^SEA^ /--^SEA^) (Fig. 2C). In agreement with the colorimetric visualization, gel electrophoresis confirmed the specificity of the three primer sets (Fig. 2A–C, lower panel, respectively). Moreover, the normal primers, SEA primers and THAI primers show a broad range of amplification temperatures from 61 to 65°C (Fig. S1), suggesting that a regular water bath could be used. The limit of detection of normal, SEA and THAI primer sets as assessed by serial dilution of known DNA samples was 1 ng, 10 ng and 1 ng, respectively (Fig. S2).

**Table 1:** List of Gap-LAMP primers.

Primer	Sequence 5' → 3'
Normal (ψα2)	
Normal-B3	GGA GAT GAG GGT TTT GG
Normal-BIP (B1c-B2)	GTG GGG CTT CAC TAG CGT TAG TCC TTC GGG TGC GGA T
Normal-F3	GCA GGT TGT CCA CG
Normal-FIP (F1c-F2)	GCA GGC TCT TCA CGG TGT ATA TTC TCA GGT GCG GGA AGT A
SEA deletion	
SEA-B3	CAC CCT CCC ACA GTT C
SEA-BIP (B1c-B2)	GAC GAC CGA GTT CCT GCG TTT TCT CTG TGT TCT CAG TAT TGG
SEA-F3	GAC GAT GCT TGC TTT GTC
SEA-FIP (F1c-F2)	GGA GGC TGG GGC AGG TAA TCA GTG TTG TAG TCA TGG C
THAI deletion	
THAI-B3	AGA AAG GTA AAG AAA TTA GAA TAG C
THAI-BIP (B1c-B2)	GGG TGA ACC GTA TGG TAT GTG TAT AGG AAG AAT AAA GCG AGA GG
THAI-F3	GAC AGA GCA AGA CTC CA
THAI-FIP (F1c-F2)	GCA GAT CCA AGG GAG CAT AAA TTG AGT GGG CAT GAG T

### Validation of α-thalassaemia 1 diagnosis by Gap-LAMP

**Figure 3:**
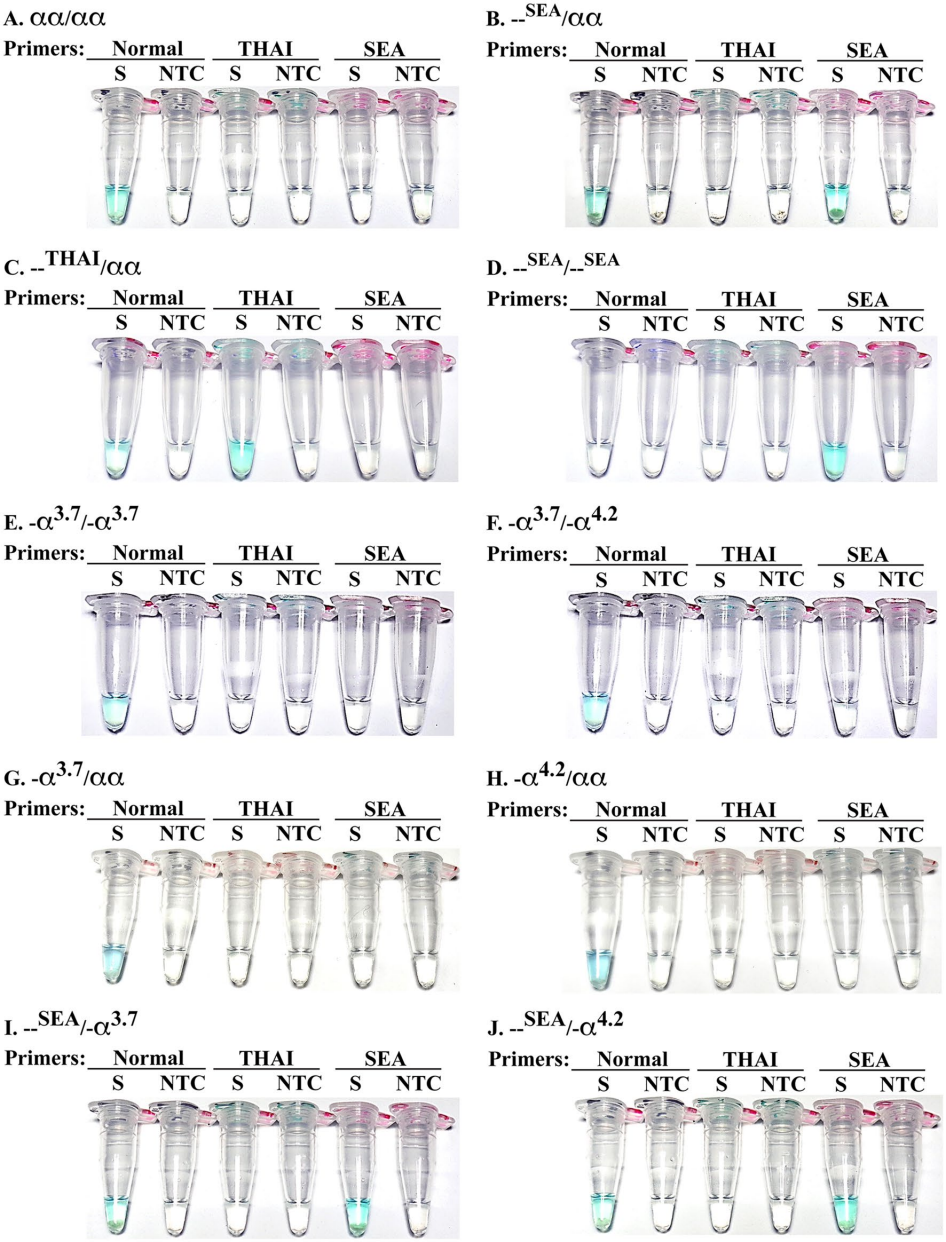
Representative of the colorimetric Gap-LAMP analysis of individuals with various α-thalassaemia genotypes. The visualization of colorimetric Gap-LAMP of (**A**) normal subject (αα/αα), (**B**) SEA trait (--^SEA^/αα), (**C**) THAI trait (--^THAI^/αα), (**D**) Hb Bart’s hydrops fetalis (--^SEA^/--^SEA^), (**E**) homozygous -α^3.7^ deletion (-α^3.7^/-α^3.7^), (**F**) compound heterozygous -α^3.7^/-α^4.2^ deletion (-α^3.7^/-α^4.2^), (**G**) heterozygous -α^3.7^ deletion (-α^3.7^/αα), (**H**) heterozygous -α^4.2^ deletion (-α^4.2^/αα), (**I**) HbH disease with -α^3.7^ deletion (--^SEA^/-α^3.7^) and (**J**) HbH disease with -α^4.2^ deletion (--^SEA^/-α^4.2^). NTC, non-template control; S, DNA sample.

Three Gap-LAMP primer sets were used for interpreting α-thalassaemia 1 genotypes via colorimetric malachite green mediated naked eye visualization. For normal subjects (αα/αα), a bright blue signal was observed only from normal primers (Fig. 3A). The SEA trait and THAI trait (--^SEA^/αα and --^THAI^/αα), signals were observed from both normal primers and primers that corresponding to the α-thalassaemia 1 deletion type (Fig. 3B–C). HbBart’s hydrops fetalis (--^SEA^ /--^SEA^) showed amplified signal only from SEA primers (Fig. 3D). α-Thalassaemia 2 (-α^3.7^ and -α^4.2^) is also prevalent in Southeast Asian populations, and inheritance leads to various α-thalassaemia genotypes and clinical manifestations of α-thalassaemia syndrome^6,7,9,10^. The homozygous and compound heterozygous α-thalassaemia 2 (-α^3.7^/-α^3.7^ and -α^3.7^/-α^4.2^) are undistinguishable by haematological parameters from heterozygous α-thalassaemia 1 (--^SEA^/αα and --^THAI^/αα), therefore, a definitive diagnosis requires DNA analysis. Herein, the Gap-LAMP technique using these sets of primers demonstrated that only normal primers could amplify homozygous and compound heterozygous α-thalassaemia 2 DNA (Fig. 3E–F). Thus, newly develop colorimetric Gap-LAMP technique could distinguish between α-thalassaemia 1 trait (--^SEA^/αα and --^THAI^/αα) and homozygous or compound heterozygous α-thalassaemia 2 (-α^3.7^/-α^3.7^ and -α^3.7^/-α^4.2^). In addition, α-thalassaemia 2 trait (-α^3.7^/αα and -α^4.2^/αα) showed amplified signal only from normal primers (Fig. 3G–H). HbH disease (--^SEA^/-α^3.7^ and --^SEA^/-α^4.2^) can be amplified by normal and SEA primers (Fig. 3I–J). This indicated that individuals carrying α-thalassaemia 1 gene (--^SEA^/αα and --^THAI^/αα) could be accurately identified.

A total of 410 genomic DNA samples from individuals carrying various α-thalassaemia genes were used for validation of the Gap-LAMP. The results were compared with those obtained from routine conventional multiplex Gap-PCR coupled with gel electrophoresis (Table 2). The normal primers amplify the ψα2-globin gene from the normal globin allele and α-thalassaemia 2 allele gave a positive blue color signal in 404 samples of normal subjects and various α-thalassaemia 2 interactions. Moreover, there was no DNA amplification from 6 samples of amniotic DNA from HbBart’s hydrops fetalis. The SEA primers gave a blue color signal from 142 samples that contained the SEA deletion, SEA trait, HbBart’s hydrops fetalis and HbH disease concordant with the conventional multiplex Gap-PCR. The THAI primers showed the blue color signal in 6 samples of THAI trait DNA samples and remained colorless in 404 samples from other genotypes. These results showed the three primer sets have 100% sensitivity, specificity and accuracy.

**Table 2:** Detection of α-thalassaemia 1 and non α-thalassaemia 1 deletion (normal) fragments by Gap-LAMP compared with the gold standard, multiplex Gap-PCR.

Gap-PCR				Gap-LAMP			Percentages (%)		
Genotype	No.	M	F	Normal	SEA	THAI	Sensitivity	Specificity	Accuracy
Normal subjects (αα/αα)	208	77	131	208	0	0	100	100	100
SEA traits (--^SEA^/αα)	122	48	74	122	122	0	100	100	100
THAI traits (--^THAI^/αα)	6	2	4	6	0	6	100	100	100
Bart’s hydrops fatalist (--^SEA^/--^SEA^)	6	0	6	0	6	0	100	100	100
Homozygous -α^3.7^ deletion (-α^3.7^/ -α^3.7^)	10	3	7	10	0	0	100	100	100
Compound heterozygous -α^3.7^ and -α^4.2^ deletion (-α^3.7^/ -α^4.2^)	3	0	3	3	0	0	100	100	100
-α^3.7^ deletion traits (-α^3.7^/αα)	21	7	14	21	0	0	100	100	100
-α^4.2^ deletion traits (-α^4.2^/αα)	20	10	10	20	0	0	100	100	100
HbH with -α^3.7^ deletion (--^SEA^/-α^3.7^)	12	1	11	12	12	0	100	100	100
HbH with -α^4.2^ deletion (--^SEA^/-α^4.2^)	2	1	1	2	2	0	100	100	100

*F* Female, *Gap-LAMP* Colorimetric Gap-loop mediated isothermal amplification, *Gap-PCR* Multiplex Gap- polymerase chain reaction, *Hb* Haemoglobin, *M* Male, *No*. Number, *SEA* The Southeast Asian type and *THAL* The Thai type.

## Discussion

α-Thalassaemia is prevalent in southern China and Southeast Asia, and several of these countries have implemented prevention and control programs for severe thalassaemias, including Hb Bart’s hydrops fetalis. In order to cover the large populations for screening couples at risk of having an affected child, accurate screening in primary care in a rural area without sophisticated equipment is needed. Herein, we report a development of a colorimetric Gap-LAMP for the visual detection of the two deletional types of α-thalassaemia 1 prevalent in southern China and Southeast Asia.

In this study, three sets of Gap-LAMP primers were designed to diagnose two common α-thalassaemia 1, the SEA deletion, and the less common THAI deletion. The method described here has not been designed to detect the -α^3.7^ and -α^4.2^ deletions of α-thalassaemia 2. Thus, a drawback of our method is that HbH disease could not be distinguished from α-thalassaemia 1 trait. However, those two thalassaemias can be simply discriminated using haematological and clinical data. The Gap-LAMP method developed here is for population screening of the most common α-thalassaemia 1 in the Asian population for prevention and control programs. The Gap-LAMP was validated in individuals carrying various α-thalassaemia genes. A limitation is that there was only a few cases of some rare genotypes due to low frequency in the population such as THAI trait (--^THAI^/αα) and HbBart’s hydrops fetalis (--^SEA^ /--^SEA^). Comparison of this new method with the multiplex Gap-PCR and gel electrophoresis-based method showed 100% sensitivity, specificity and accuracy of the three primer sets.

Diagnosis of the SEA deletion allele by LAMP has been reported using pH-sensitive dyes or fluorescence dyes^18,20,25^. Recently, methods for detecting the THAI deletion by LAMP using pH-sensitive dyes was reported^20^. The drawback of the pH-sensitive dyes is the requirement of optimal pH for the best results and the difficulty of distinguishing positive readout, changing the color of phenol red from pink to orange. The limitation of fluorescent dye is the need of develop of fluorescence under illumination by ultraviolet light. This study used malachite green dye, which provides a notable advantage as malachite green is mixed prior to amplification. The results can be observed and determined by naked eye visualization in a closed system without opening the reaction tubes or post-amplification handling, thereby reducing the risk of cross-contamination. The malachite green signal recognition is highly sensitive and enables visual discrimination of results without costly specialized equipment. The positive samples turned light blue, whereas the negative samples turned from green to colorless after the Gap-LAMP reaction.

In summary, the Gap-LAMP using malachite green dye was found to be rapid, sensitive and reliable for screening of two common α-thalassaemia 1 that are prevalent in Asian populations. The technique allows naked eye visualization and is achieved within 60 min using a general water bath or heating block, obviating the need for a thermocycler or post-amplification processing. The technique described in this study could be applied for large-scale diagnosis of α-thalassaemia 1 in primary care facilities and rural areas.

## Materials and methods

### DNA samples

Genomic DNA from a total of 410 known α-thalassaemia genotype included 208 normal subjects (αα/αα), 128 α-thalassaemia 1 heterozygotes (--^SEA^/αα and --^THAI^/αα), 13 α-thalassaemia 2 homozygotes (-α^3.7^/-α^3.7^) or compound heterozygous (-α^3.7^/-α^4.2^), 41 α-thalassaemia 2 heterozygotes (-α^3.7^/αα and -α^4.2^/αα), 14 HbH disease (--^SEA^/-α^3.7^ and --^SEA^/-α^4.2^), obtained from thalassaemia screening of individuals older than 18 years of age, and 6 HbBart’s hydrops fetalis (--^SEA^ /--^SEA^), obtained from cord blood samples. The criteria for α-thalassaemia diagnosis was first screening by red blood cell indices and haemoglobin analyses with cutoff mean corpuscular volume (MCV) lower than 80 fL and HbA_2_ less than 3.5 %^26^. The deletional α-thalassaemia mutations were then analysed by conventional multiplex Gap-PCR followed by gel electrophoresis as previously described^12^. This study was performed in accordance with the Helsinki declaration and was approved by the Mahidol University Institutional Review Board (approval number MU-IRB 2008/301.2001 and MU-CIRB 2017/129.0707). Written informed consent was obtained from all individual participants in this study or their parents.

### LAMP primer design

Three specific primer sets for detection of the two α-thalassaemia 1 (SEA and THAI primers) and non α-thalassaemia 1 deletion (normal primers) were designed based on the principle of LAMP primers as described in a previous study by Notomi T and colleagues^13^ (Table 1). Normal primer set, the ψα2-globin (*HBAP2* or *HBM*) was selected for use as representative of non α-thalassaemia 1 deletion as the gene located in the overlap deleted sequence of the two deletional α-thalassaemia 1 and not located in the deleted region of α-thalassaemia 2 (Fig. 1A). The amplicon size was 278 bp, encompassing intron 1 to exon 2 of ψα2-globin gene (Fig. S3A). To detect the two deletional α-thalassaemia 1, SEA and THAI types two primer sets were designed according to the Gap-PCR principle by using the primers flanking the deleted breakpoint previously characterized (Fig. 1B–C)^27,28^. The SEA and THAI primer sets amplify 297 and 335 bp across the SEA and THAI breakpoint junction, respectively (Fig. S3B and S3C).

### Loop-mediated isothermal amplification method

Three sets of Gap-LAMP primers were prepared in separate reaction tubes. Gap-LAMP reaction was performed in a total volume of 25 μL. The reaction was containing 0.2 μM for each outer primer (B3 and F3), 0.8 μM for each inner primer (BIP and FIP), 1 M betaine (Sigma-Aldrich, St. Louis, MO), 1× isothermal amplification buffer (20 mM Tris-HCl, 50 mM KCl, 10 mM (NH_4_)_2_SO_4_, 2 mM MgSO_4_ and 0.1% Tween 20), 2 mM MgSO_4_ (New England Biolabs, Ipswich, MA) and 0.9 mM dNTP for SEA primer set or 1.1 mM dNTP for normal and THAI primer sets. The reaction mixture was pre-heated at 95°C for 15 min and cooled on ice. Then, 8 U *Bst* 2.0 WarmStart DNA Polymerase (New England Biolabs) and 0.16 μg/μL malachite green (Sigma-Aldrich) were added and followed by incubating at 64°C for 60 min. After the incubation step, reaction tubes were placed under visible light for 15 min. Positive reaction with amplified DNA products showed light blue color solution. In contrast, the negative reaction without amplification showed a colorless solution.

### Statistical analysis

Sensitivity, specificity and accuracy of visualization Gap-LAMP were calculated against results of Gap-PCR as standard method^12^.

## Supplementary Information

Supplementary Information.

## Data Availability

All data related to this study can be obtained on reasonable request to the corresponding author S.S., while all the analysed data were included in this published article and its supplementary information files.
