# Supplementary material for: Diagnosis of α-thalassaemia by colorimetric gap loop mediated isothermal amplification
Source: Sci Rep. 2023 Jun 13;13:9612. doi: 10.1038/s41598-023-36676-2 (PMC10264369; doi:10.1038/s41598-023-36676-2)
Supplement: Supplementary file 1 — Supplementary Information. [file 41598_2023_36676_MOESM1_ESM.pdf]

Supplementary Figures

Diagnosis of α-thalassaemia by colorimetric Gap Loop Mediated Isothermal Amplification

Worakawee Chumworathayee[1,2,3], Thongperm Munkongdee[2], Nattrika Buasuwan[2], Pornthip Chaichompoo[4] Saovaros Svasti[2,5,*]

[1]Graduate Program in Molecular Medicine, Faculty of Science, Mahidol University, Bangkok, Thailand; [2]Thalassemia Research Center, Institute of Molecular Biosciences, Mahidol University, Nakhon Pathom, Thailand; [3]Division of Biology, Faculty of Science and Technology, Rajamangala University of Technology Thanyaburi, Pathumthani, Thailand; [4]Department of Pathobiology, Faculty of Science, Mahidol University, Bangkok, Thailand; and [5]Department of Biochemistry, Faculty of Science, Mahidol University, Bangkok, Thailand

Number of supplementary figures: 3

***Correspondence:** Saovaros Svasti, Ph.D.
Thalassemia Research Center, Institute of Molecular Biosciences,
Mahidol University, Salaya, Nakhon Pathom 73170 Thailand.
Phone: +662-889-2558; Fax: +662-889-2559
e-mail: saovaros.sva@mahidol.ac.th, stssv@yahoo.com

Supplementary Figures

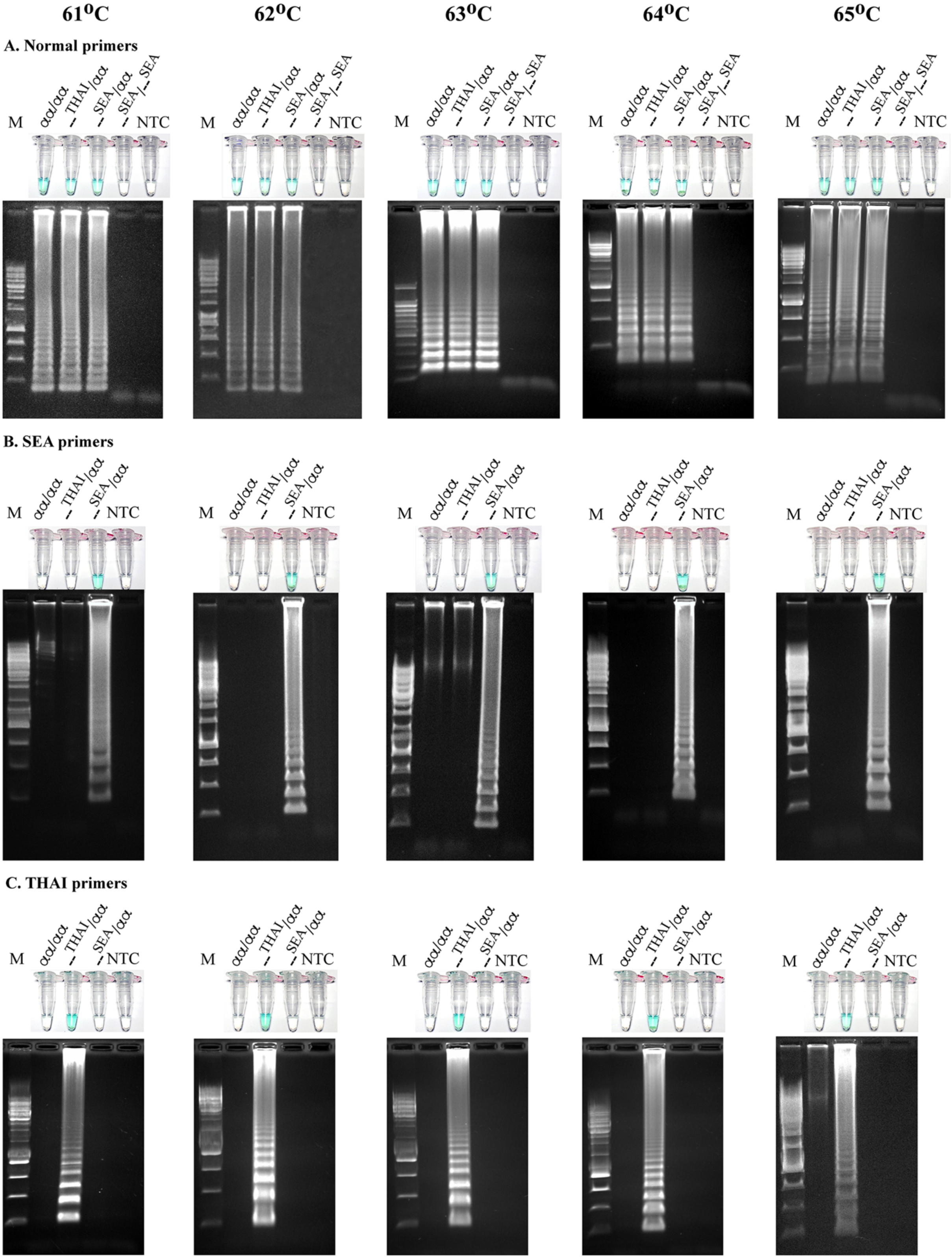

Figure S1. Wide temperature range of Gap-LAMP reaction. The optimal Gap-LAMP reaction temperature was determined by amplification of genomic DNA from normal subject

(αα/αα), α-thalassaemia 1 traits THAI deletion ($--^{THAI}$/αα) and SEA deletion ($--^{SEA}$/αα), at 61-65°C using (A) normal primers, (B) SEA primers and (C) THAI primers. Blue color from malachite green colorimetric detection in agreement with agarose gel electrophoresis analysis show specific amplification of the three Gap-LAMP primer sets. M, 1 kb DNA ladder; NTC, non-template control.

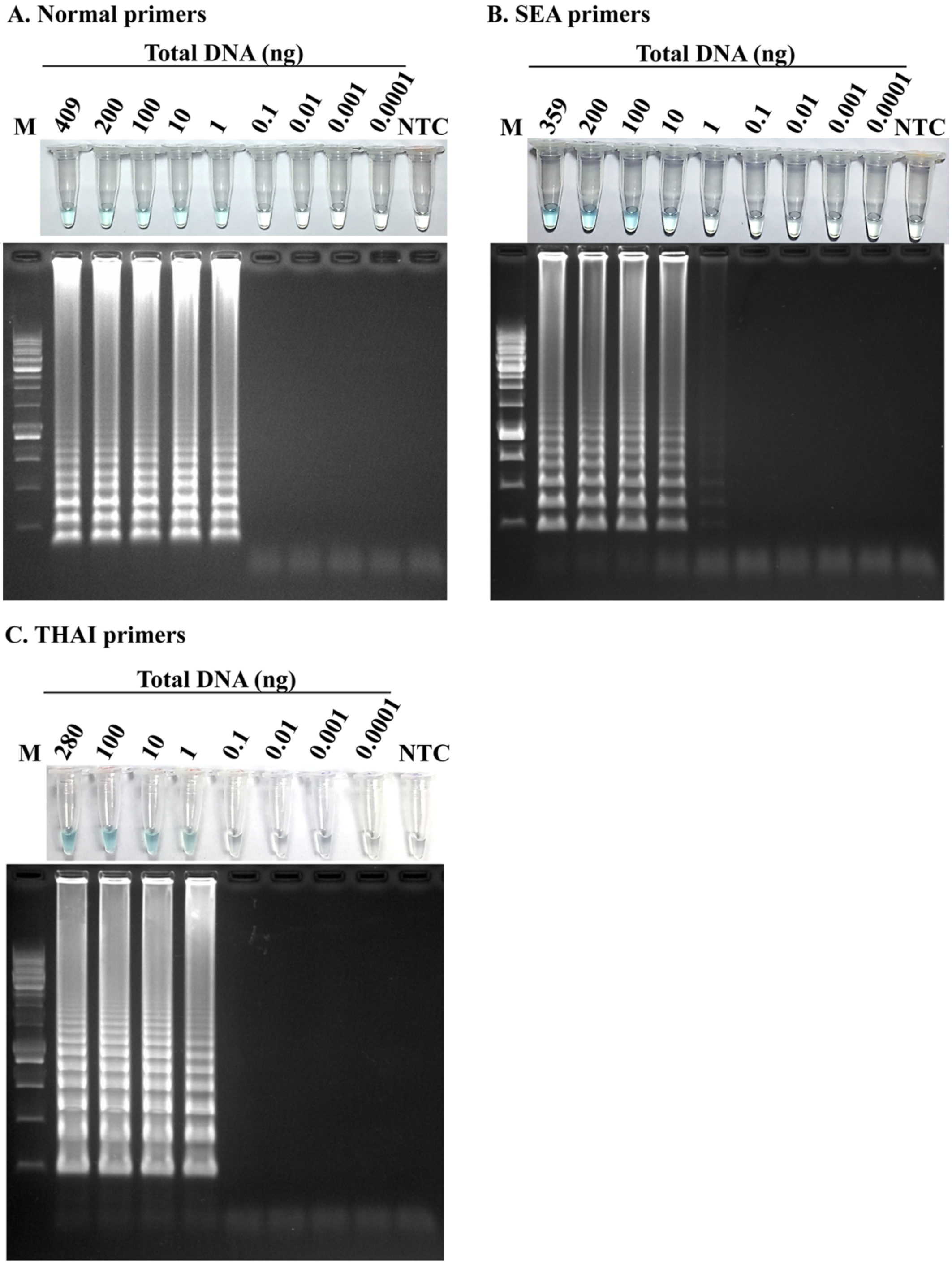

Figure S2. Limitation of detection of Gap-LAMP. The limit of Gap-LAMP detection of each Gap-LAMP primer sets (A) normal primers, (B) SEA primers and (C) THAI primers was performed by amplification of vary amount of genomic DNA from undiluted to 0.0001 ng DNA from normal subject ($\alpha\alpha/\alpha\alpha$), α-thalassaemia 1 traits SEA deletion ($--^{SEA}/\alpha\alpha$) and THAI deletion ($--^{THAI}/\alpha\alpha$), respectively. The limit of detection of normal primers, SEA

primers and THAI primers was 1 ng, 10 ng and 1 ng, respectively. M, 1 kb DNA ladder; NTC, non-template control.

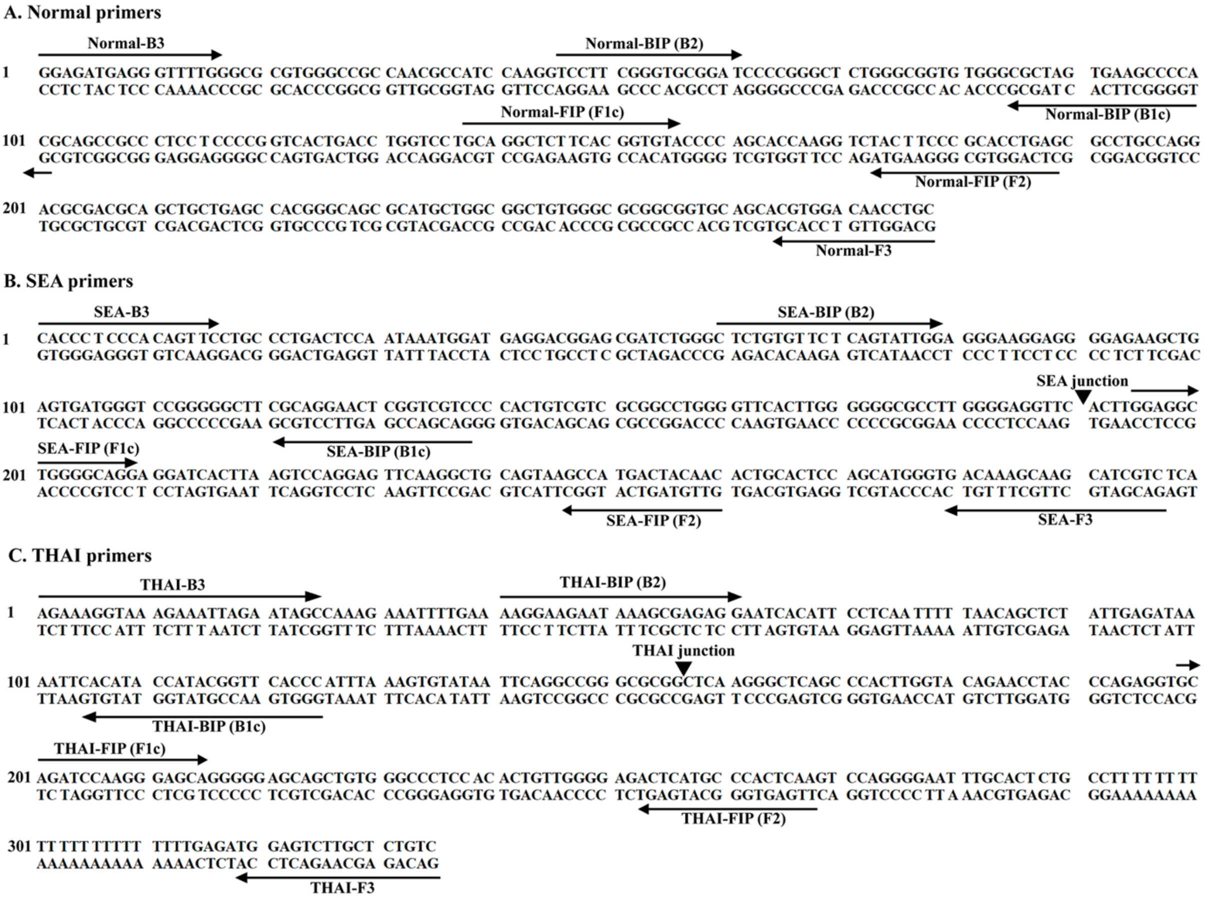

Figure S3. **Position of the Gap-LAMP primers.** Nucleotide sequences indicate six sequences recognized by the four Gap-LAMP primers for detection of (A) Normal (ψα2-globin), (B) SEA deletion and (C) THAI deletion. Arrows head indicate breakpoint junction. Underline arrows indicate direction of primers sequence.
